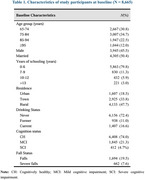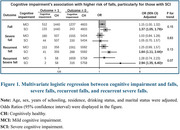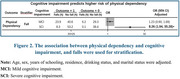# Is cognitive impairment associated with higher risk of falls and subsequent physical dependency? A nationwide longitudinal study in China

**DOI:** 10.1002/alz70860_102679

**Published:** 2025-12-23

**Authors:** Mingzhi Yu, Yuling Jiang, Longbing Ren, Wenjian Zhou, Shaojie Li, Yang Hu, Yifei Wu, Jing Wu, Zhenhan Yu, Yutong Wang, Yejing Zhao, Jie Zhang, Yanyan Zhao, Jing Li, Yao Yao

**Affiliations:** ^1^ School of Public Health, China Centers for Health Development Studies, Peking University, Beijing, Beijing, China; ^2^ Peking University, Beijing, Beijing, China; ^3^ China Centers for Health Development Studies, Peking University, Beijing, Beijing, China; ^4^ School of public health, Peking university, Beijing, Beijing, China; ^5^ Department of Geriatrics, Institute of Geriatric Medicine, Beijing Hospital, National Center of Gerontology, Chinese Academy of Medical Sciences, Beijing 100005, China., Beijing, Beijing, China

## Abstract

**Background:**

The association between cognitive impairment among older adults and risks of falls and physical dependence still remains unclear.

**Method:**

In this longitudinal study, we aim to investigate whether cognitive impairment increases the risk of falls, recurrent falls, and subsequent physical dependency in older adults. We used data from the 8th and 9th waves (2018‐2021) of the Chinese Longitudinal Healthy Longevity Survey (CLHLS), a nationally representative cohort of adults aged 65 and older in China. Of 14,779 participants aged 65 and older surveyed in 2018, 8,665 were followed up in 2021 and included in the analysis. Falls were assessed through self‐reports over the past 12 months, with severe falls defined as requiring medical treatment. Physical dependency was defined as any difficulty in functional mobility or being bedridden. Participants were categorized by their Mini‐Mental State Examination (MMSE) scores: cognitively healthy (CH, MMSE: 25–30), mild cognitive impairment (MCI, MMSE: 10–24), and severe cognitive impairment (SCI, MMSE: 0–9). Multivariate logistic regression was used to examine associations between cognition and the risk of falls and physical dependency.

**Result:**

At baseline, 6,408 (74.0%) participants were CH, 1,845 (21.3%) had MCI, and 412 (4.7%) had SCI. Falls were reported by 1,694 (19.5%) participants, with 662 (7.6%) reporting severe falls. At follow‐up, 2,085 (24.1%) participants reported falls, and 731 (8.4%) reported severe falls. Cognitive impairment was associated with a significantly higher risk of falls, particularly for those with SCI (OR for SCI vs. CH: 1.37 [95%CI 1.05–1.79] for falls). Among baseline fallers, cognitive impairment was linked to recurrent falls (OR for SCI: 1.64 [95%CI 1.11–2.44]) and recurrent severe falls (OR for SCI: 2.94 [95%CI 1.35–6.40]). Cognitive impairment also predicted higher risk of fall‐related physical dependency (OR for SCI: 8.26 [95%CI 1.94–35.28]).

**Conclusion:**

Cognitive impairment significantly increases the risk of falls and subsequent physical dependency in older adults, adversely affecting late‐life quality and placing substantial burdens on family caregivers. Targeted interventions to prevent falls and mitigate dependency in this vulnerable population are urgently needed.